# The Experiences of Late-diagnosed Women with Autism Spectrum Conditions: An Investigation of the Female Autism Phenotype

**DOI:** 10.1007/s10803-016-2872-8

**Published:** 2016-07-25

**Authors:** Sarah Bargiela, Robyn Steward, William Mandy

**Affiliations:** 1 Research Department of Clinical, Educational and Health Psychology, UCL, Gower Street, London, WC1E 6BT UK; 2 Centre for Research in Autism and Education, UCL Institute of Child Health, 55-59 Gordon Square, London, WC1H 0NU UK

**Keywords:** Autism spectrum conditions (ASC), Autism spectrum disorder (ASD), Diagnosis, Female autism phenotype

## Abstract

We used Framework Analysis to investigate the female autism phenotype and its impact upon the under-recognition of autism spectrum conditions (ASC) in girls and women. Fourteen women with ASC (aged 22–30 years) diagnosed in late adolescence or adulthood gave in-depth accounts of: ‘pretending to be normal’; of how their gender led various professionals to miss their ASC; and of conflicts between ASC and a traditional feminine identity. Experiences of sexual abuse were widespread in this sample, partially reflecting specific vulnerabilities from being a female with undiagnosed ASC. Training would improve teachers’ and clinicians’ recognition of ASC in females, so that timely identification can mitigate risks and promote wellbeing of girls and women on the autism spectrum.

## Introduction

Autism spectrum condition (ASC), also known as ‘autism spectrum disorder’ (ASD)^1^ is a neurodevelopmental syndrome characterised by difficulties with social reciprocity, social communication, flexibility and sensory processing (American Psychiatric Association [APA] 2013). People with ASC are at risk of a range of emotional, behavioural, social, occupational and economic difficulties (e.g., Howlin and Moss 2012) . The timely identification of ASC can mitigate some of these risks and improve quality of life, for example by identifying needs and appropriate interventions, increasing access to services, making others less judgemental of the person with ASC and their parents, reducing self-criticism, and helping to foster a positive sense of identity (Hurlbutt and Charmers 2002; Portway and Johnson 2005; Ruiz Calzada et al. 2012; Russell and Norwich 2012; Wong et al., 2015).

Compared to males, females are at substantially elevated risk of their ASC going undiagnosed: their difficulties are frequently mislabelled or missed entirely (Lai and Baron-Cohen 2015). This is shown by the observation that in non-referred samples there are between two and three males for each female with ASC (e.g., Constantino et al. 2010; Kim et al. 2011; Zwaigenbaum et al. 2012); whereas in clinical samples ascertained from ASC services, the male-to-female ratio is usually four-to-one or higher (e.g., Fombonne 2009). Thus, many females who, if skilfully assessed, would meet full diagnostic criteria for ASC, never receive a diagnosis and the help that, potentially, comes with it. Even when females with ASC are identified, they receive their diagnosis (and associated support) later than equivalent males (Giarelli et al. 2010). Furthermore, compared to males, females require more severe autistic symptoms (Russell et al. 2010) and greater cognitive and behavioural problems (Dworzynski et al. 2012) to meet ASC criteria, and teachers underreport autistic traits in their female pupils (Posserud et al. 2006). This gender bias has serious consequences for the health and wellbeing of girls and women with ASC, and has been identified by the autism community as a key problem to be addressed by research (Pellicano et al. 2014).

One proposed explanation of the ascertainment bias against females with ASC is that there is a female autism phenotype; a female-specific manifestation of autistic strengths and difficulties, which fits imperfectly with current, male-based conceptualisations of ASC. (APA 2013; Hiller et al. 2014; Lai et al. 2015; Mandy et al. 2012). There is an emerging evidence-base to support the existence of this female autism phenotype. For example, in line with the reports of clinicians and people with ASC, there is empirical evidence that girls and women with ASC show higher social motivation and a greater capacity for traditional friendships than do males with ASC (Head et al. 2014; Sedgewick et al. 2015). Furthermore, compared to equivalent males, females with ASC are less likely to have externalising behaviours, such as hyperactivity/impulsivity and conduct problems, and are more vulnerable to internalising problems, such as anxiety, depression and eating disorders (Mandy et al. 2012; Huke et al. 2013); and consistently score lower on measures of repetitive and stereotyped behaviour (Van Wjingaarden-Cremers et al. 2014).

Nevertheless, research into ASC gender differences is at an early stage, and there is currently no definitive account of the female autism phenotype that could be used to inform efforts to reduce the ascertainment bias against girls and women with ASC (Lai et al. 2015). Findings on gender differences in core diagnostic social and communication symptoms have been inconclusive, with there being no clear picture as to whether, compared to males with ASC, females with ASC either show greater (e.g., Hartley and Sikora 2009), lesser (e.g., McLennan et al. 1993) or equal social difficulties (e.g., Mandy et al. 2012). Also, it is uncertain whether the consistent observation that females score lower than males for repetitive behaviours reflects genuinely lower levels of these traits, or if female-typical repetitive behaviours do not register on current measurement tools (Van Wjingaarden-Cremers et al., 2014). Further, it has been suggested that a key feature of the female autism phenotype is a capacity to ‘camouflage’ social difficulties in social situations (e.g., Kenyon 2014). However, despite promising initial investigations (e.g., Baldwin and Costley 2015; Cridland et al. 2014; Mandy and Tchanturia 2015; Rynkiewicz et al. 2016) further work is required to operationalize the construct of camouflaging, to prepare the ground for the development of measures that could be used in quantitative investigations.

These uncertainties about the nature of the female autism phenotype reflect, in part, two key methodological challenges to doing research in this area, which have constrained the validity of findings to date. First, most studies have investigated participants with ASC ascertained from autism clinics [e.g., see reviews by Lai et al. (2015) and Van Wjingaarden-Cremers et al. (2014)]. This has the effect of excluding the very participants most relevant to the research, namely females who have been missed by clinical services because their ASC exemplifies the female autism phenotype. Second, males and females have tended to be compared on gold-standard, well-established measures of ASC symptoms. For most ASC research this would be a methodological strength, but when investigating gender differences it is potentially problematic. Such measures have been developed and validated with largely male samples, and may lack sensitivity to the female autism phenotype. Therefore, there is a need for measures that are demonstrably sensitive to autistic features as they present in females as well as males (Lai et al. 2015). Both of the methodological problems described above would have the effect of underestimating ASC gender differences. A further consideration is that no research to date has sought to examine directly how the female autism phenotype can lead to a situation whereby a girl or woman meets criteria for ASC, but is missed by professionals. Such investigations will be informative for those seeking to improve diagnostic practice to reduce gender-based inequities in ASC care.

Given the above, in order to advance the study of ASC gender differences we conducted a study with three key features. First, we aimed to investigate directly not only the nature of the female autism phenotype, but also how it impacts upon risk of a girl and/or woman’s ASC going unrecognised. Second, we recruited women with ASC whose autistic difficulties had gone unrecognised in infancy, childhood and early adolescence. We reasoned that such late-diagnosed individuals would be more likely to exemplify elements of the female autism phenotype that are under-represented in samples of those identified in a timely fashion, and can provide insights into how such characteristics led to them being missed by clinical services. This approach is supported by the recent finding that the gender ratio in adult ASC clinics is lower (two males to one female) than in child and adolescent services (five males to one female), suggesting that later-diagnosed samples are most likely to include a representative sample of females with ASC (Rutherford et al. 2016). Third, we took an inductive (i.e., data-driven) approach, conducting a qualitative investigation. Our aim was not to test hypotheses about the female autism phenotype by formally comparing males and females. Rather, we sought to generate new ideas and deepen understanding of key concepts, such as ‘camouflaging’ (Barker and Pistrang 2015). This work is designed to yield novel, well-defined hypotheses about the female autism phenotype to guide future quantitative investigations; and to promote the development of measures that capture female as well as male manifestations of ASC.

Framework Analysis (Ritchie et al. 2003), widely applied in health research to generate theory and promote the development of new measures, was used to analyse qualitative data from in-depth interviews in order to address the following questions:

1. What is the nature of the female autism phenotype, as experienced by late-diagnosed women with ASC?
2. How does the female autism phenotype influence young women’s experiences of diagnosis, misdiagnosis and missed diagnosis?
3. How do late-diagnosed women with ASC adapt in response to the challenges they face?

## Methods

### Participants

Participants were 14 women with ASC. Women were eligible to participate in the study if they met the following inclusion criteria: (1) aged between 18 and 35 years; (2) diagnosed with ASC by a certified professional (psychiatrist, clinical psychologist) in the UK National Health Service; (3) ASC diagnosis was received in late adolescence or adulthood (aged 15 years or older); (4) ASC diagnosis had been received within 10 years of study participation; (5) living in the United Kingdom; (6) without an intellectual disability, as indicated by having an IQ above 70. Age and IQ limits for the sample were set to limit group heterogeneity, as these variables likely condition the experiences of women with ASC. Seventeen women contacted the researcher asking to participate in the study. Three did not meet eligibility criteria: one was outside of the qualifying age range (51 years) and two lived outside of the UK (USA and Australia).

Participant characteristics are shown in Table 1. At the time of the study, seven of the participants reported being able to support themselves through paid employment. One had a profession, but was signed off work due to mental health problems. The three full-time students were supported by research grants or family. The remaining participants did volunteer work or full-time parenting. The mean age of the participants was 26.7 years, (SD = 2.3). All their ASC diagnoses had been made since 2004, and the mean age of diagnosis was 21.3 years (SD = 4.8). Thirteen had received their diagnosis from a specialist autism service, having never previously been assessed for ASC. One participant was diagnosed after assessment in a Child and Adolescent Mental Health Service, also without any previous history of autism assessment. The research team did not retrieve clinical records to double-check the veracity of self-reported diagnoses, but every participant scored above cut-off on the AQ-10. All participants had estimated IQs above 70 on the Wechsler Test of Adult Reading.

**Table 1:** Characteristics of the sample

Participant	Age at time of interview	Age at diagnosis	IQ	AQ-10 (cut-off = 6)	Employment
P01	23–26 years	19–22 years	122	9	Illustrator
P02	23–26 years	15–18 years	115	6	Full time Student
P03	27–30 years	27–30 years	110	8	Health Professional
P04	22–26 years	15–18 years	113	10	Support Worker
P05	22–26 years	22–25 years	122	10	Volunteer
P06	27–30 years	23–26 years	124	9	Full time Student
P07	19–22 years	19–22 years	124	9	Fine Artist
P08	22–26 years	19–22 years	85	10	Health Professional
P09	27–30 years	19–22 years	108	8	Administrator
P10	27–30 years	23–26 years	117	9	Volunteer
P11	27–30 years	27–30 years	108	8	Full-time mother
P12	27–30 years	19–22 years	115	8	Full-time Student
P13	23–26 years	19–22 years	92	10	Volunteer
P14	27–30 years	23–26 years	110	9	Athlete

Precise age and age at time of diagnosis are not given to protect participant confidentiality

AQ-10 = 10 item version of the Autism Spectrum Quotient

### Procedure

Participants were recruited via existing contacts within the research team and through adverts placed on social media sites frequented by women with ASC. Information sheets were provided to eligible participants at least 24 hours before consent was sought, and only those who subsequently gave full informed consent joined the study. All 14 participants completed quantitative measures of IQ, depression, anxiety and autistic traits (see Measures) in order to ‘situate the sample’, i.e. to give the reader a sense of who the participants where, to inform thinking about transferability of the findings (Barker and Pistrang 2015). Nine interviews were conducted face-to-face, four participants were interviewed via Internet videoconferencing, and one interview was completed on the telephone. Videoconference and telephone interviews were conducted in order to avoid excluding participants who did not want a face-to-face interviews, due to anxiety, sensory issues and/or a reluctance to engage in direct social interaction.

### Measures

#### Semi-structured Interview

Data were collected using a semi-structured interview schedule developed specifically for this study by the research team, via a process of consultation with women with ASC, expert clinicians and researchers. Thus, the topics covered in the interview reflected the research aims, previous research, clinical insights into the female autism phenotype and the priorities of members of the autism community. In line with guidelines for semi-structured interviews, it was designed to be used flexibly, thus allowing the interviewer freely to follow the participant’s line of response, maximising the chances of collecting valid, in-depth data from people with atypical social communication (Smith 1995). The interview began by asking women to tell the story of how they were diagnosed with ASC, including exploration of their sense of how their gender had impacted upon this. It then moved to consideration of interests, social relationships, sensory experiences and mental health. The final questions concerned the participants’ perceptions of ASC gender differences. A copy of the interview schedule is available by request from the first author (SB).

#### Autism Quotient-10 (AQ-10)

This ten-item, self-report, brief version of the Autism Spectrum Quotient was used to confirm clinical diagnosis, and to give an approximate measure of ASC severity (Allison et al. 2012).

#### General Health Questionnaire-12 (GHQ-12)

The GHQ-12 is a twelve-item, brief version of the GHQ-60. It is used as a screening device to assess the respondent’s current mental state (Goldberg and Williams 1988) and is a reliable and valid measure of severity of psychological difficulties (Goldberg et al. 1997). The GHQ-12 has a maximum score of 36. For young adults, 24 has been established as an optimal cut-point for identifying mental health difficulties (Makowska et al. 2002). Participants were asked to complete the measure for the researchers to gain an overview of the mental wellbeing of the sample.

#### Hospital Anxiety and Depression Scale (HADS)

The HADS is a fourteen-item self-assessment scale used to detect depression (seven items) and anxiety (seven items) over a 1-week period (Snaith 2003). Participants are asked to respond to statements that best describe their state of mind, with higher scores indicating greater symptom severity. Both the depression and anxiety subscales have a maximum score of 21, with scores of 8 and above being indicative of clinically severe difficulties.

#### Wechsler Test for Adult Reading (WTAR)

The WTAR is a neuropsychological assessment tool, which provides a reliable, valid and low intensity estimate of intelligence (Holdnack 2001). In this study, researchers used the WTAR in order to offer a quick estimate of verbal IQ, to establish whether participants were able to easily verbalise their experiences, to situate the sample and to check that participants met the inclusion criterion of not having an intellectual disability.

### Data Analysis

All interviews were transcribed verbatim, and Framework Analysis was applied to the data (Ritchie and Spencer 1994; Ritchie et al. 2003). This method involves following a structured sequence of steps in order to systematically identify themes within qualitative data. Framework Analysis was chosen as it is a widely used and transparent method of qualitative analysis that allows researchers to generate new theory from data whilst focusing their inquiries on pre-determined research objectives. We adhered to guidelines for good practice in qualitative research (Mays and Pope 2000; Pope et al. 2000; Stiles 1999) by conducting the following credibility checks, to ensure that interpretations of the data were sound and fair. First, to avoid relying upon a single researcher’s perspective, a consensus approach (Barker and Pistrang 2005) was employed: SB took the lead in analysis but all three authors regularly discussed themes until, eventually, they derived a final set of themes and subthemes. Second, during this process, WM audited the framework against transcript data (Elliott et al. 1999). Finally, respondent credibility checks were conducted to promote testimonial validity (Barker and Pistrang 2005), whereby the framework was sent to participants for their feedback, to ensure it reflected their experiences.

## Results

### Anxiety, Depression and Wellbeing in the Sample

All fourteen participants completed the HADS and GHQ-12. The mean HADS-A (anxiety) score was 13.5 (SD 3.7, range 7–20), which lies above the recommended clinical cut-off. All but one participant scored above the cut-off score. The mean HADS-D (depression) score was 5.3 (SD 4.8, range 1–15), below the clinical threshold, with three participants scoring in the clinical range. The mean GHQ-12 score was 15.4 (SD 4.4, range 9–27), below the cut-off indicating mental disorder. Three participants scored in the ‘distress’ range and a further two fell in the ‘severe’ range, indicative of severe psychological difficulties at the time of interview.

### Qualitative Analysis: Themes and Sub-themes

Relevant data in the transcripts were organised into themes and subthemes, with the final thematic framework presented in Table 2. Four themes, comprising nineteen subthemes, were identified. Quotes were labelled as ‘P’ followed by a unique identifier for each participant (Table 1). The four major identified themes are as follows: (1) ‘You’re not autistic’, which recognises common barriers to gaining a diagnosis as a woman; (2) ‘Pretending to be normal’ which identifies strategies that young women employed when trying to fit in with their peers; (3) ‘Passive to assertive’ explores how passivity and social naivety impact on young women with ASD and how they have learnt to be assertive; and (4) ‘Forging an identity as a young woman with ASC’ outlines common difficulties associated with being female with a social communication disorder and the protective role played by special interests.

#### “You’re not autistic”

This theme included reported experiences of autistic difficulties being ignored and misunderstood, perceived reasons for this, and beliefs about the implications of having received a late diagnosis. Almost all the young women reported having experienced one or more mental health difficulty, with anxiety, depression and eating disorder being the most commonly reported. Most participants commented that health professionals treating them had not noticed their symptoms might be related to ASC:

“Four to five years of depression and anxiety treatment…years of talking therapy…and not once did anyone suggest I had anything other than depression”. (P05)

Even when participants had begun to suspect that they might have ASC, for example after suggestions from friends or family members, when they approached health professionals, their concerns were often dismissed. After having researched ASC and decided to pursue a diagnosis from their family doctor (in the UK called a ‘general practitioner’ [GP]), five participants reported that their GPs had dismissed their concerns and did not offer further assessment. Others reported being misdiagnosed:

“You go to your doctor…and you get diagnosed with multiple personality disorder which is completely opposite to what you are.” (P07)

In contrast, there were two exceptional cases of a speedy diagnosis: both young women had been immediately referred for assessment after presenting to their GPs who had recognised signs of autism in their behaviours.In most cases, young women thought that their delay in receiving a diagnosis was partially due to a lack of professional knowledge of how autism presents specifically in females:

“When I mentioned the possibility to my psychiatric nurse she actually laughed at me…I asked my mum, who was a GP at the time…if she thought I was autistic. She said, ‘Of course not’. At the time, a good 10 years ago now, there just wasn’t much information about how girls presented, and from what she knew, I was nothing of the sort.” (P05)

Participants also suggested that a stereotype that people with ASC all have very severe and overt social and communication problems added to professionals’ reluctance to diagnose females who showed some capacity, albeit superficial, to socialise with others. Young women also felt that *‘Rain Man’* (P03) stereotypes, which incorrectly assume that ASC is always associated with savant skills and with an interest in mathematics and science, had delayed their diagnoses.

“I’ll always remember my special needs teacher saying I’m too poor at maths to be autistic.” (P04)

Teachers were the other significant professionals who young women had experienced as having had little knowledge of female ASC. When reflecting on their school years, young women reported that their passive and compliant behaviours had often been misinterpreted as being ‘shy’ or ‘good’. Several recalled being regarded as the “teacher’s pet” (P04) or the ‘model pupil’. In contrast to their good behaviour in school, these women recalled having had regular emotional ‘meltdowns’ at home after school:

“I was unbearable with my mother, but at school I was perfect” (P09).

Some young women suggested that as their quiet and passive behaviours were seen as socially acceptable for girls, they had gone unnoticed, and proposed that had they been more disruptive they might have been noticed sooner.

“The reward for trying hard to be normal was to be ignored because you were acting normal and I look at stories online of kids who were going off the rails and I think, I should have just burnt more cars” (P09)

Interview data suggested that during secondary school, teachers’ misinterpretations of autistic girls’ behaviours changed. A number of young women said that they had been told they were ‘rude’ or ‘lazy’ after they had made social *faux pas*, due to their misunderstanding of social rules:

“I was often accused of being rude when I had absolutely no intention of being so…he [a teacher] started saying I wasn’t trying and that I was a waste of his time.” (P04)

Other poignant examples of being misunderstood came from young women who had been bullied. Upon complaining to their teachers, they recalled that *they* were blamed and been told to try and ‘act more normal’.

“When I was being bullied, I was told not to antagonise these girls and actually I was only antagonising them by being myself.” (P03)

Most of the interviewed young women were diagnosed between the ages of 20 and 30, and many shared how they thought a delayed diagnosis had been detrimental to their wellbeing and education:

“I think women tend to be diagnosed later in life when they actually push for it themselves…when you’re a child, you don’t realise that you’re anxious and depressed… [that] your education is going to suffer because of that and I think that if I had known, and if people had helped me from earlier on, then life would’ve been a whole lot easier.” (P07)

Women also talked about their emotional reactions to having a late diagnosis, and shared their regret and anger at having “tried to be good” *(P09)* for so long, and as a result, been missed. One young woman felt that knowing about her diagnosis could have protected her in risky social situations:

“Had I known about Asperger’s, I think I’d have known that I’m more suggestible…and I might not have ended up in the situations that I did.” (P14)

**Table 2:** - Framework analysis, including frequency of themes

Theme	Subtheme	Frequency
“You’re not autistic”	Labelled with non-autistic diagnoses	12
	Unhelpful professional stereotypes of autism	12
	Quiet at school, so went unnoticed: ‘I should have burnt more cars’	6
	Misunderstood, unsupported or blamed by teachers	8
	The costs of a late diagnosis	8
Pretending to be ‘normal’	‘Wearing a mask’	8
	Learning social behaviours from TV, books and magazines	6
	Social mimicry	5
	The costs of masking	5
Passive to assertive	Please, appease, avoid conflict	7
	Entrapment in abusive relationships or risky situations	8
	Victim of sexual abuse	9
	Learning to be assertive	8
Forging an identity as a woman with ASD	Societal pressures: what is expected of young women	11
	Friendships: uncertainty and intensity	12
	Hard to be friends with neurotypical girls	8
	Easier to be friends with boys	7
	Friendships and support online	7
	Interests define identity and self confidence	7

#### Pretending to be Normal

Interestingly, most women reported that, whilst in childhood teachers did not notice their difficulties, other children were very sensitive to their differences. This theme includes such experiences as being seen by peers as ‘different’ and subsequent attempts to ‘fit in’. The subthemes describe the strategies that women learnt from various media and other people and young women’s reflections on the costs of pretending to be someone else.

Socialising as part of large groups was reported as challenging by all the young women interviewed. To cope, many described ‘wearing a mask’ or taking on a certain ‘persona’, when in specific social situations:

“I honed something of a persona which was kind of bubbly and vivacious, and maybe a bit dim, because I had nothing to say other than adult novels. So I cultivated an image, I suppose, that I brought out to social situations as my partner’s girlfriend, that was not ‘me’.” (P09)

Masking was also used to hide autistic traits in order to appear ‘normal’. One woman described using her ‘mask’ as a ‘double-bluff’ technique to openly joke about an aspect of her behaviour that her peers might have labelled as autistic:

“I’ll mask if I act weird which is typical of ASC, I’ll make a joke about it.” (P02)

Another woman described using alcohol as a way to “free me up to maintain my neurotypical mask” (P03) in situations where she needed to pretend to be interested in conversation topics, such as television programmes that she did not like. Many women described actively learning how to ‘mask’ from different media sources including characters on television, magazines, books on body language and novels:

“They’d have the right behaviour for certain things, so ‘If you want this, you should do this’.” (P02)

Another woman learnt phrases and facial expressions from fictional literature in order to manage more unpleasant situations, such as bullying.

“When I was being bullied, there’s this book by Ellen Montgomery and the character Emily, whenever somebody is horrible to her…she just looks at them, and because of her expression they go away.” (P04)

Social mimicry was another strategy used in social situations. However, young women reported that mimicry was automatic and unconscious, in contrast to their willed and conscious masking behaviours.

“I honestly didn’t know I was doing it [social mimicry] until I was diagnosed, but when I read about it, it made perfect sense. I copy speech patterns and certain body language.” (P05)

Some young women had noticed that they would quickly pick up accents from other people and suggested that this may have been an unconscious attempt to create an increased sense of familiarity when socialising with new people:

“I automatically mimic what other people are doing, what people are saying, how people say things, I went on [Girl Guide] camps…and I would come back with strong accents. But I can’t consciously put on an accent…my way of coping is that I mimic.” (P06)

Despite having acquired superficially adaptive strategies for coping in social situations, young women also identified some significant costs related to ‘masking’ and ‘mimicking’. Many had found that the effort required to process consciously people’s behaviours and later act them out, was exhausting:

“It’s very draining trying to figure out everything all the time, everything is more like on a manual, you’ve got to use one of those computers where you have to type every command in.” (P01)

Others reported having felt confused about their identity as a result of pretending to be someone else, indeed some had “acted neurotypical” (P07) so convincingly that at times they had doubted whether they had ASC.

#### Passive to Assertive

Many participants described experiences of victimisation, and related this to their autistic difficulties. When participants considered their experiences of victimisation, their accounts often included narratives of passivity and assertiveness. They described how their perceived passivity, which they linked to their ASC, had led to unhealthy relationships and high-risk situations. One participant described feeling the need to “please, appease and apologise - do what you’re told” (P03) in order to feel accepted and receive affection. When describing her response to unwanted requests for sex within a relationship another said:

“I almost feel pressured by society to do it because you get told this is what is expected of you to make to be a good girlfriend and you think, if I don’t do it, then I am not fulfilling my duties (P08).”

Many participants also described going to great lengths to avoid conflict. One woman reflected that for many years she had considered any form of verbal disagreement as *‘having a fight’* (P03). In other cases, women who had previously avoided conflict talked about having over-asserted themselves at times, resulting in lasting damage to friendships: “When it finally comes out I can be a bit too blunt” (P10). However, there were two exceptions to women seeing themselves as being passive. One woman in particular made it clear that she had never appeased if she knew something was wrong.

There was a shockingly high incidence (9 of 14 participants) of sexual abuse reported in this sample. Half of the accounts of sexual abuse were reported to have happened in relationships. These young women spoke about feeling obliged or “gradually being pestered’” (P14) into having sex, as something they felt was expected of them in a girlfriend role. Another commented that arguments would “end up in us having sex even if I didn’t want to” (P11). Three young women reported being raped by people they did not know. In one particularly distressing case a young woman was groomed by a peer at the bidding of three older men. The data yielded a number of interlinked reasons as to how young women had become entrapped in situations where their safety and rights were compromised. First, the role of social mimicry was considered:

“There’s potential for you copying a guy’s flirtatious behaviour without realising that’s what you’re doing (P05).”

Second, many women reported finding it difficult to ‘read’ other people’s intentions, and so struggled to understand if a man was just being friendly or was sexually attracted to them. Third, in contrast to their neurotypical female peers who could share skills to keep themselves safe, young women with ASC reported feeling isolated as teenagers and so lacking a point of reference from which to develop strategies for staying safe. Fourth, some women reported that their experiences of peer rejection left them ‘desperate’ for acceptance, which in turn made them more vulnerable to exploitation:

“Because we don’t sense danger and can’t. That’s one reason, I think you not reading people to be able to tell if they’re being creepy, you’re that desperate for friends and relationships that if someone is showing an interest in you, you kind of go with it and tend not to learn from others’ safety skills.” (P07)

Fifth, young women’s uncertainty regarding social rules was also mentioned as contributing to risk of abuse. For example, some had not known that they *could* say ‘no’ when they had wanted to refuse sex or other people’s advances. At times when they had known they could refuse, young women reported that they had not known *how* to say ‘no’ or how to leave a situation until it was too late. Others talked about being trapped in unhealthy relationships:

“I kept trying to break up with him and whenever I did he would say I didn’t know my own feelings…I was at my wit’s end I felt so trapped (P04).”

Despite narratives of passivity and accounts of abuse, a number of women also talked about an increase over time in their ability to be assertive. Young women who had reported difficult interpersonal experiences were able to later reflect on and describe how they had been manipulated. As a result, many described having learnt to read others’ intentions better and used this knowledge to leave situations where they felt uncomfortable: “[there were] times when guys pushed for it, so I just walked away” (P05). Other women used skills they had actively learnt, such as “a guide to being assertive” (P02) provided by a counsellor and skills learnt in their jobs.

Four women explained how they had used their diagnosis of Asperger’s as a tool to give them more confidence asserting their opinion. Women commented that before having a diagnosis they would have “just kept quiet” (P10) but now they were able to ask people for clarification or explanations when they were unsure of a situation. One participant who had previously used her diagnosis as a means of explaining difficulties, now no longer felt obliged to provide an explanation and felt confident enough to simply say ‘no’.

#### Forging an Identity as a Woman with ASD

The final theme concerns young women’s perceptions of social gender stereotypes that they had felt pressured to, struggled to, and at times, refused to fulfil. Related to this, their experiences of navigating and negotiating friendships are also explored.

Young women’s opinions varied regarding gender-stereotypical roles. Whilst some openly rejected gender-based theories of behaviour: “[I don’t] really accept the validity of gender stereotypes” (P04) or ‘status quo’ behaviours, for example, dating someone their own age: “He’s 50, err so what, such conventions never bothered me before” (P07), other women were more tentative. Indeed, some young women described having tried to fulfil socially expected roles: “caring, nurturing, ‘looking out for everyone’, kind of role” (P08), and many had experienced a sense of lost identity, where they felt “I knew that I wasn’t being me” (P10) when trying to play ‘the wife’ or ‘girlfriend’.

Young women discussed how ASC affected their ability to form friendships. One difficulty raised by three women was ‘defining a friend’. All three had to think consciously of situations in order to decipher if someone was a friend:

“this person sort of dealt with this and was still my friend afterwards, [so] they must be good friends (P11)”

Another challenge young women experienced was not knowing how to navigate the nuances of friendly interactions:

“Not knowing what was expected of me, not being able to pick up on when to provide support or how often to get in touch (P09).”

When reflecting on previous school friendships, a number of young women commented that the main areas of conflict had often been related to wanting the exclusive attention of a particular (and often, only) friend.

“A lot of my problems came about with them having other friends that I didn’t like or I didn’t get on with…I didn’t really want to share them.” (P10)

Young women also reflected on people who they found it easier, and more difficult, to be friends with. Eight participants reflected on their experiences of trying to form friendships with neurotypical women and had often found it hard to manage what they perceived were socially expected skills of a woman. During teenage years, many of their female peers were noticeably more sophisticated in their social abilities: “girls…socially are a lot quicker” (P02). The young women had also found gossip and competition amongst females difficult to navigate.

“The gossip…in women that can be quite hard…if they’re talking about someone sometimes it’s hard to know whether they are… [being] mean…you know you worry that if you say the wrong thing with other women that you’re going to be talked about behind your back.” (P10)

One woman commented that at times, girls would notice someone who was vulnerable and “they’ll generally put her down” (P02). Another remembered feeling intimidated by neurotypical teenage girls and had experienced rejection for being seen as “one iota different from them”. (P03). In contrast, a number of young women said they felt more at ease in their friendships with males. This was not thought to be related to biological sex, but to society ‘allowing’ men to be more straightforward, and this being a communication style that suited women with ASC better:

“I just feel so much more comfortable with men because they’re more, you can take them at face value and its not that fear of them judging you or having alternative motives and thoughts and they kind of say things straight.” (P07)

Several women spoke of the importance of friendships that they had made or maintained using online media. Friendships with other ‘Aspie’ women from online forums were particularly important. One woman described her friends as a: “gang of fellow Aspie women who I think of as my family” (P10). Some had found that their visits to online forums had increased their pride and confidence in having a diagnosis:

“It’s a difference not a disorder…it was really helpful because it made me feel good about myself (P02).”

Other women used blogs as a way of hearing other women’s stories and sharing their own, and as a result felt accepted and understood by others who have been through similar experiences.

“Something that I really appreciate about having the diagnosis is actually being in this club now where people talk about their experiences and having so many echoes of my own.” (P03)

The use of an online platform had made communication easier for some young women. For example, in normal face-to-face communication, one would be expected to ‘read’ body language, tone of voice and facial expression, so “If all we have is typing for each other, then it’s completely equal” (P10). Women talked about being able to express themselves more clearly when they didn’t have the pressure and anxiety to respond immediately, as with a face-to-face conversation. Further, use of messaging was also an easier and less awkward medium to express difficult emotions and access support from their friends. Despite the previously mentioned challenges with establishing friendships, it is notable that most of the young women had a small number of consistent friends from school or university. Finally, young women’s interests appeared to be a defining feature of their identities and an important part of their *raison d’etre*. Indeed, for those whose interest was their full-time occupation it defined them as people, gave them a focus in life and provided a sense of personal well-being:

“I was very obsessed, and still am, with creative writing and that kind of provides the entire focus of my life…I would say I forge most of my identity on that and the degree…they allow me to express myself in different ways, they form the basis of my identity.” (P09)

Reported interests varied enormously, from animals to international boat racing, from sexuality, physics and The Middle East to autism and events organising and provided them with structure and a sense of achievement:

“It’s very good…for my self-belief, to see that that I can do something that’s recognised by other people as beneficial and productive (P04).”

Therefore, instead of relying on common social norms, such as sociability or motherhood, to define themselves, young women formed their identities through their special interests.

## Discussion

We interviewed 14 women with ASC who had received their diagnosis in late adolescence or early adulthood, in order to learn more about the female autism phenotype and how it impacts upon the recognition of ASC. We took an inductive (i.e., data-driven), qualitative approach in order to generate new ideas and to deepen understanding of existing concepts. Our aim is that this work will contribute to future quantitative investigations of the female autism phenotype by generating novel hypotheses and informing the development of appropriate measures.

One element of the female autism phenotype that is elucidated by our analyses is the phenomenon of ‘camouflaging’, or ‘pretending to be normal’ (Holliday-Willey 2014). Efforts to camouflage were widespread but not universal in this sample. Participants spoke of making a deliberate effort to learn and use ‘neurotypical’ social skills, sometimes describing this as ‘putting on a mask’ (Baldwin and Costley 2015; Cridland et al. 2014). Our analyses suggest that the development of such neurotypical personas may rely on concerted and prolonged autodidacticism based on, for example, careful observation of peers, reading novels and psychology books, imitating fictional characters, and trial and error learning in social situations. In addition, we also identified unconscious elements to camouflaging that warrant further investigation, whereby women reported their social behaviour being copied from others around them without even realising they were mimicking in this way.

Another observation about camouflaging is that it was reported to be associated with various disadvantages. These included a sense of exhaustion and confusion about the individual’s true identity. Furthermore, as is discussed below, some women believed that a tendency to mimic others and prioritise fitting in above their own needs had led them to be manipulated and abused by others; and had caused others not to notice their needs for help. The current analyses do not in any sense provide a definitive picture of camouflaging. Rather, we intend that the account of camouflaging given in the present paper, and other research reports of the phenomenon (e.g., Baldwin and Costley 2015; Cridland et al. 2014), be used to derive a precise and coherent conceptualisation of this construct, including both its conscious and unconscious elements. This operationalization of camouflaging can then form the basis for the design of a measure which can be used in quantitative investigations to address questions such as: ‘how widespread is camouflaging amongst people with ASC?’; ‘are there age and gender differences in camouflaging?’; ‘what are the costs and benefits of camouflaging?; ‘what could educational establishments and workplaces do to minimise the camouflaging that people with ASC have to do?’. We believe that this camouflaging measure, at least initially, should rely on self-report, as by its very nature, camouflaging behaviour is often not obvious to observers.

Our data suggest that some of the challenges of being a female with ASC do not come directly from the individual’s autistic difficulties; but rather reflect how these difficulties play out within a culture that has specific expectations for females. Some women in the sample reported a conflict between their desire to accept their autistic selves, and perceived pressures to fulfil traditional gender roles. For example, participants described feeling pressure to play certain traditional feminine roles (the wife, the mother, the girlfriend), and finding this incompatible with how they wanted to live as a person with ASC. Further, in line with findings for adolescent girls with ASC (Cridland et al. 2014), several women in our sample reported that their autistic social communication difficulties made it difficult for them to join and enjoy female peer groups, which they perceived as being more subtle and less forgiving of *faux pas* than male social groups. There is emerging evidence that having ASC increases a person’s chances of not identifying with their assigned-at-birth gender (Strang et al. 2014), or of rejecting a binary gender identity (Kristensen and Broome 2015). Although no one in the current study reported being at odds with their gender as assigned at birth, our finding that some women experience a conflict between a feminine and autistic identity suggests one potential influence on the elevated rates of gender dysphoria and non-binary gender amongst natal females with ASC.

As discussed above, ASC in females is more likely to be identified late, to be mis-labelled or not to be recognised at all. Based on our investigation of the experiences of females whose ASC went unidentified in childhood and early/mid adolescence, we hypothesise that this phenomenon reflects both: (1) specific features of the female autism phenotype; and (2) characteristics of the systems that are designed to identify and help people with ASC. Reflecting this, we propose a model of the bias against recognising female ASC, for testing in future quantitative, deductive (i.e. theory-driven) studies. In terms of individual characteristics, we suggest that females who are especially invested in and skilled at camouflaging are at greater risk of their ASC going undetected (see also Baldwin and Costley 2015). Further, we hypothesise that the female tendency to have internalising (e.g., anxiety, depression) but not externalising (e.g., hyperactivity/impulsivity, conduct problems) difficulties is also a risk factor for non-detection of ASC, first because children with these problems tend not to be disruptive at school as so are less likely to be referred by teachers for clinical help, and, second, because internalising difficulties sometimes over-shadow an underlying ASC diagnosis. In addition we propose that greater social motivation (Sedgewick et al. 2015) and better non-verbal communication (Rynkiewicz et al. 2016), both characteristics of the female autism phenotype, should be investigated as factors that can lead professionals to rule out ASC as a possible diagnosis when a females with autistic difficulties presents for assessment.

We also propose that the knowledge and assumptions of teachers, GPs, mental healthcare workers and other professionals contribute to the diagnostic bias against females with ASC. Participants reported that some professionals showed biases that females could not have ASC, and/or had conceptions of ASC that did not encompass more subtle, female-typical presentations. Inevitably, such biases seem to have been especially common amongst those who are not specialist in assessing neurodevelopmental disorders, but who are nevertheless influential gate-keepers to relevant services for people with ASC, such as teachers (Baldwin and Costley 2015; Cridland et al. 2014) and GPs (family doctors).

Our findings also highlight the costs of the bias against female ASC diagnosis. In line with studies of adults with ASC (see Howlin and Moss 2012), internalising difficulties were common amongst participants. All but one reported clinically severe anxiety, and levels of distress were elevated, emphasising the importance of identifying women with ASC in order to provide support, including for co-occurring emotional difficulties. Most participants (8 of 14) explicitly stated that their lives would have been easier had they received their diagnosis earlier in life. Pre-diagnosis, they recalled being misunderstood, with their autistic difficulties often labelled in very negative terms by peers and adults, for example as laziness or wilful defiance. In line with findings for mixed-gender samples of adults with ASC (e.g., Hurlbutt and Chalmers 2002) most participants had experienced the eventual receipt of their diagnosis as helpful. Some stated that it had fostered a sense of belonging in a group of like-minded people, and that this had promoted a more positive sense of self. The Internet and social media appear to be especially important for enabling the existence of such communities. Thus, our findings suggest that another cost of missed diagnosis is that it denies people with ASC the opportunity to benefit from identifying with the autism community.

To our knowledge, this is the first study to specifically investigate the experiences of late-diagnosed females with ASC, and we believe that our inductive, in-depth approach has generated some new insights into the female autism phenotype and its impact upon risk of missed diagnosis. Nevertheless, it is important to think critically about the nature of the sample we recruited and how this affects the transferability of our findings to females with ASC beyond the study sample (Barker and Pistrang 2015). First, although our sample experienced significant mental health difficulties, especially anxiety, that are in line with expectations for the general population of adults with ASC they are notably ‘high-functioning’, in the sense that they mostly had above-average IQs and a greater proportion were in work or education than is commonly reported for adults with ASC (Howlin and Moss 2012). Therefore it will be important in future to investigate experiences of women across the full range of severity of the autism spectrum. Second, this investigation took place in the UK. It is likely that the impact of the female phenotype on risk of being missed will be different in other countries with different healthcare systems, and this should be investigated. Third, our focus on late-diagnosed females made us less likely to uncover ‘success stories’ in terms of timely recognition and support. In future, the study of girls with ASC who were recognised in early childhood could be useful for providing pointers towards good clinical and educational practice in this area, and could generate different ideas about the female phenotype to those of the current study. Fourth, older women and those diagnosed even later than those in the current study may have distinct experiences and needs, and this warrants direct investigation.

Our focus exclusively on females and use of qualitative methodology meant that we have not made direct comparisons between males and females. It is possible that some of our findings are not specific to females, and also apply to males with ASC, especially those who have average and above intelligence, fluent speech and who received their diagnosis late. The current research can provide a foundation for future quantitative investigations which make formal gender comparisons, to learn the extent to which males with ASC camouflage, are vulnerable to sexual exploitation, are liable to have their difficulties missed by professionals, and find their autistic characteristics clash with their attempts to conform to gender norms. Furthermore, the testing of ideas generated by the current study in representative samples of females with ASC will serve to formally address uncertainties about their transferability, described above.

The current study has sought to generate hypotheses about the presentation and challenges faced by women who meet diagnostic criteria for ASC, but who were not picked up in childhood. It should be noted that there is another group of females who have severe autistic-like difficulties (i.e., difficulties with social reciprocity, social communication, flexibility and sensory processing), but who do not actually meet diagnostic criteria for ASC (Dworzynski et al. 2012). It is unclear whether such individuals should be included in studies of the female autism phenotype. One argument is that, because they do not qualify for a diagnosis, they cannot be considered representative of females with ASC. The contrary position is that they really do have ASC, but fail to meet criteria because current diagnostic criteria are insensitive to their more female-typical presentation. Currently we lack clinically applicable biomarkers for ASC (Mandy and Lai 2016), so there is no definitive way to resolve the question of whether women with autistic-like difficulties who do not meet ASC criteria exemplify the female autism phenotype, or are actually experiencing a non-autistic condition. Nevertheless, the study of such individuals will be informative about whether their difficulties are appropriately captured by the ASC label. Such work should address: (1) whether, despite not meeting specific diagnostic criteria, their social, sensory and flexibility difficulties fall within the broad conceptual domains of ASC (Lai et al. 2015); (2) whether their associated impairments are clinically severe; and (3) whether the pattern of their associated impairments is comparable to that seen in females who do meet ASC diagnostic criteria. It will also be instructive to take a longitudinal approach, to investigate whether some females experience later onset of clinically-severe autistic difficulties, failing to meet diagnostic criteria as children but then going on to qualify for an ASC diagnosis in adolescence or adulthood.

## Conclusions

In their account summarizing the priorities of autism research based on interviews with people with ASC, (Pellicano et al. 2014) stressed the need to understand why women with ASC ‘slip through the net’ and to identify ways to counter this gender-based inequity in current clinical practice. Our findings suggest that the attainment of these goals will require several courses of action. First, research to define the female autism phenotype must include the development of measures of camouflaging, so that this phenomenon can be studied quantitatively, increasing understanding of its prevalence and effects on diagnosis and wellbeing. Second, levels of knowledge about ASC and training needs of a range of healthcare and educational professionals, including those who do not specialize in ASC, should be investigated. This would lay the ground for the development of training programs about the female autism phenotype, to improve recognition and referral to appropriate services. Finally, our finding, which is echoed elsewhere in the scientific (Cridland et al. 2014) and autobiographical literature, that having ASC increases the risk for females of being sexually abused, requires immediate attention (e.g., Steward 2014). More research is needed to test the ideas suggested by our participants about why they were vulnerable to exploitation; and this should inform specific training programmes for females with ASC, to help them stay safe.

## Footnotes

1 We use the term ‘autism spectrum conditions’ (ASC) as a direct synonym for the DSM-5 term ‘autism spectrum disorder’ (ASD). This is in accordance with views of members of the autism community, to be more respectful of neurodiversity. Our use of ‘ASC’ aims to convey that people on the autism spectrum show differences that include strengths as well as difficulties.
